# Genetic Variation between *Biomphalaria alexandrina* Snails Susceptible and Resistant to *Schistosoma mansoni* Infection

**DOI:** 10.1155/2013/160320

**Published:** 2013-06-26

**Authors:** Suzanne M. F. El-Nassery, Iman F. Abou-El-Naga, Sonia R. Allam, Eman A. Shaat, Rasha F. M. Mady

**Affiliations:** ^1^Medical Parasitology Department, Faculty of Medicine, University of Alexandria, Alexandria, Egypt; ^2^Medical Biochemistry Department, Faculty of Medicine, University of Alexandria, Alexandria, Egypt

## Abstract

Much effort has been made to control schistosomiasis infection in Egypt. However, enduring effects from such strategies have not yet been achieved. In this study, we sought to determine the genetic variability related to the interaction between *Biomphalaria alexandrina* snails and *Schistosoma mansoni*. Using RAPD-PCR with eight (10 mers) random primers, we were able to determine the polymorphic markers that differed between snails susceptible and resistant to *Schistosoma mansoni* infection using five primers out of the eight. Our results suggest that the RAPD-PCR technique is an efficient means by which to compare genomes and to detect genetic variations between schistosomiasis intermediate hosts. The RAPD technique with the above-noted primers can identify genomic markers that are specifically related to the *Biomphalaria alexandrina/Schistosoma mansoni* relationship in the absence of specific nucleotide sequence information. This approach could be used in epidemiologic surveys to investigate genetic diversity among *Biomphalaria alexandrina* snails. The ability to determine resistant markers in *Biomphalaria alexandrina* snails could potentially lead to further studies that use refractory snails as agents to control the spread of schistosomiasis.

## 1. Introduction

Schistosomiasis is the second most common parasitic cause of mortality in tropical countries, second only to malaria, and has been a target for increased control by the World Health Organization [1]. Great effort has been made to control the transmission of the disease. However, there is little evidence that the prevalence of the disease has decreased globally. Indeed, it continues to spread to new geographic areas [2]. Of the countries in which neglected tropical diseases are prevalent, Egypt is one of the many counties that have suffered greatly under the burden of tropical diseases, including schistosomiasis [3].* Schistosoma* infection is one of the most significant public health problems facing the country.

Human infection with *Schistosoma mansoni* is closely related to the existence of its intermediate snail host of the genus *Biomphalaria*. *Biomphalaria alexandrina* is the only snail host in Egypt [4, 5]. This snail has extended its distribution from the Nile Delta and is now present throughout the country along the tributaries of the Nile. By 1979, *B. alexandrina* had colonized the Nile from the Delta to Lake Nasser [6]. Recently, *B. alexandrina* snails have invaded the water sources in some reclaimed areas, which has resulted in *Schistosoma* infection in previously uninfected populations and will eventually lead to an increase in schistosomiasis transmission throughout Egypt [7]. The situation may be further complicated by the increasing number of irrigation projects whose goal is to improve the Egyptian economy along with the increasing mobility of infected agricultural workers who migrate to neighboring areas [8, 9]. Schistosomiasis is a specific target for control by the Egyptian Ministry of Health, international agencies, and by the World Bank. This has been done via the mass administration of the medication praziquantel to human populations in conjunction with the use of niclosamide for snail control. However, schistosomiasis control has not yet been achieved [10]. With the appearance of praziquantel resistance among schistosomes [11] and the absence of an available vaccine for use [12], the use of alternative control strategies is necessary. The high degree of specificity of schistosomes for their intermediate snail host led Coelho et al. [13] to suggest a control strategy based on the use of snails resistant to parasitic infection as biological competitors of existing susceptible snails in endemic areas. However, the application of this strategy requires an in-depth understanding of the role of genetics in the interaction between parasites and snails. 

Newton [14], a pioneer in the study of the genetics of *Biomphalaria glabrata*, considered the susceptibility or resistance of a snail to infection to be a heritable characteristic. Resistance is found to be a dominant character in many *Biomphalaria spp.* However, some loci of susceptible snails could carry a certain amount of resistance alleles [15, 16]. The increase in the number of inherited resistant genes could lead to an increased immune response to the parasite and decrease the degree of susceptibility to infection [16–18].

Resistance in *Biomphalaria* snails has been found to be age dependent. In *B. glabrata*, the resistant phenotype in juvenile snails is controlled by four genes, each of which has multiple alleles, while in adulthood, there is only a single dominant gene that determines this trait [19, 20]. In *B. tenagophila*, two dominant genes determine resistance [17]. The susceptibility and resistance of *B. alexandrina*, as with other *Biomphalaria spp*., were found to be heritable characteristics, with the resistance trait being dominant [16, 21]. However, the number of genes that determine this characteristic has not yet been identified.

One of the factors limiting progress in understanding the molecular genetics of *B. alexandrina* relative to other *Biomphalaria* species has been the lack of specific sequence information for the application of polymerase chain reaction (PCR). An alternative powerful and highly applicable technique that does not require prior sequence information is the random-amplified polymorphic DNA-polymerase chain reaction (RAPD-PCR). Unlike conventional PCR-based analyses, this approach uses oligonucleotide primers of an arbitrary sequence to start DNA strand synthesis under conditions of low stringency. Each primer allows for the amplification of specific fragments that are randomly distributed throughout the genome of the snail, thus allowing for unbiased comparison [22, 23]. Many investigators have used this technique to distinguish between susceptible and resistant phenotypes in different *Biomphalaria *species due to its high applicability [24–27]. In this study, we sought to determine the genetic variability related to the susceptibility and resistance of *B. alexandrina* snails to *S. mansoni* using the RAPD-PCR technique with different primers.

## 2. Materials and Methods

### 2.1. Snails Stocks

All snails originated from water channels in the Alexandria governorate (Egypt). They were maintained for one year in our laboratory under suitable environmental conditions in glass aquaria containing snail-conditioned water, and they were fed lettuce leaves and calcium carbonate. The parasite strain was originally obtained from shedding cercariae from naturally infected* B. alexandrina* snails collected from Alexandria water courses. Maintenance of *S. mansoni* life cycles was conducted between the snails and Swiss strain albino mice. Separation of *Schistosoma *resistant and susceptible snail stocks was performed according to the method described by Zanotti-Magalhães et al. [28], which is as follows: 100 juvenile snails (four to six mm in diameter) were used. Each snail was exposed for four hours to eight freshly hatched miracidia of *S. mansoni*. Susceptibility was monitored weekly from four up to 10 weeks postexposure through cercariae shedding which was favored by lamp light. Nonshedding snails were reexposed individually to eight miracidia/snails. Any snails in which the cercareia were observed were considered infected, and those that did not shed cercariae after two exposures were considered uninfected. The infection rate was calculated.

Snails that remained uninfected after two exposures were isolated and reared singly for self-reproduction under laboratory conditions. Their progeny was the resistant stock. Snails that yielded high infection rates were isolated and reared singly for self-reproduction, and their progeny was the susceptible stock. Thirty days post infection, six snails from each stock were used in the present study.

### 2.2. Molecular Analysis

#### 2.2.1. DNA Extraction

Snail tissue (both susceptible and resistant) was dissected, and the head-foot of each snail was preserved in absolute ethanol for the extraction of high molecular weight DNA [4, 29]. EZ-10 spin column genomic DNA extraction kit for animal tissue (Bio Basic Inc., Canada) was used. Thirty mg of the snail tissue was placed in a 1.5 mL centrifuge tube, and 300 *μ*L of Animal Cell Lysis Solution and 20 *μ*L proteinase K were added. The mixture was incubated at 55°C with occasional vortexing until the tissue was completely lysed (at least three hours) and cooled to room temperature. The tube was mixed by vortex for 20 seconds and then centrifuged at 14500 ×g for five minutes. 300 *μ*L of the supernatant was then pipetted into an EZ-10 spin column, and 300 *μ*L of AB buffer solution was added. The preparation was mixed well and allowed to sit for two minutes. Centrifugation was completed at 1600 ×g for two minutes, and the flow through was discarded. 500 *μ*L of the wash solution was added, and spinning was conducted at 6400 ×g for one minute. Washing was repeated, and the flow-through was discarded. Spinning was completed at 10060 ×g for an additional minute to remove the residual amount of the wash solution. The column was placed into a clean 1.5 mL microfuge tube, and 45 *μ*L elution buffer (2.0 mM Tris-HCl pH 8.0–8.5) was added to the central portion of the membrane in the column. The tube was incubated at 50°C for two minutes. Spinning was conducted at 10060 ×g for one minute to elute DNA from the column.

The quantity of DNA was measured spectrophotometrically by UV absorption at A260 (1.0 OD unit is equivalent of 50 *μ*g). The quality of genomic DNA was assessed via an analytical 0.7% agarose gel.

#### 2.2.2. DNA Amplification by RAPD-PCR

The genotypes of the resistant and susceptible strains of *B. alexandrina* snails were determined with different arbitrary 10 mer primers using the RAPD-PCR technique according to a method described by Simpson et al. [30] and Abdel-Hamid et al. [25] with some modifications. The technique was performed in a reaction volume of 25 *μ*L using 25 ng genomic DNA of each sample, 50 pmoL of each primer, 10X Taq DNA polymerase buffer including MgCl_2_, 0.2 mM dNTPs, and five unit/*μ*L Taq DNA polymerase (Promega Co., USA).

Eight 10 mer primers were tested for their ability to differentiate between susceptible and resistant strains of *B. alexandrina* to *S. mansoni*. The primers used were as follows: OPA-01    (5′-CAGGCCCTTC-3′), OPA-02 (5′-TGCCGAGCTG-3′), OPA-06 (5′-GGTCCCTGAC-3′), OPB-18 (5′-CCACAGCAGT-3′), OPC-11    (5′-AAAGCTGCGG-3′), OPD-10 (5′-GGTCTACACC-3′), OPD-18 (5′-GAGAGCCAAC-3′), and OPM-04 (5′-GGCGGTTGTC-3′) (Operon Technologies Inc. CA, USA). The primers OPA-01, OPA-02, OPA-06, and OPM-04 have been used in previous studies in which specific polymorphic markers were detected between resistant and susceptible strains of different snail species to *S. mansoni* infection. They were used in *B. glabrata* [24, 26, 31, 32], *B. tenagophila* [25], in both *B. glabrata *and *B. tenagophila* [33], and *B. alexandrina *[21]. To our knowledge, the remaining random primers had not yet been tried in *Biomphalaria spp*. 

The lyophilized primers were purchased from Eurofins (mwg/operon). They were reconstituted by adding sterile water to a final concentration of 100 pmoL/*μ*L distributed in aliquots and stored at −20°C. The amplification conditions were as follows [21]: the tubes were transferred to the thermal cycler where they were subjected to one cycle of initial denaturation at 95°C for five minutes, then 40 cycles of 95°C for one minute, 30°C for one minute, 72°C for one minute followed by a final extension cycle at 72°C for 10 minutes.

#### 2.2.3. Agarose Gel Electrophoresis and Staining

The PCR products were separated on 2% agarose gel stained with ethidium bromide, visualized on a UV Transilluminator, and photographed by Gel Documentation System (Alpha Imager M1220, Documentation and Analysis System, Canada). 

#### 2.2.4. Analysis of Polymorphisms on Agarose Gels

The genetic variability of the susceptible and resistant strains was determined by analysis of the electrophoretic profiles of the bands visualized on the gels. The identification of polymorphic bands was based on the comparison of the band patterns on the same gel for the susceptible and the resistant strains, and only those detected in all individuals of the same strain were considered polymorphic [24, 33]. The similarity coefficient was calculated as described by Dice [34] (Table 1).

## 3. Results

The susceptibility or resistance of *B. alexandrina* snails to *S. mansoni* infection was studied starting four weeks postinfection and continuing weekly thereafter for up to 10 weeks after miracidia exposure. The obtained results showed that 68% of the examined snails (100 snails) were susceptible, while 32% of the snails were resistant. 

In this study, the susceptible and resistant snail lines were selected on the basis of well-characterized resistance/susceptibility phenotypes after uninfected snails were exposed to *S. mansoni* miracidia twice. The RAPD-PCR method was used to differentiate between susceptible and resistant snail lines of *B. alexandrina*. A total of eight primers were tested, and polymorphic markers were obtained with five of them (OPA-02, OPB-18, OPC-11, OPD-10, and OPD-18). We found that with these primers, the method was reliable for the identification of stable genomic markers between the two phenotypes, that is, the susceptible and resistant traits within the same snail species. The other three primers, OPA-01, OPA-06 and OPM-04, did not produce any results.

**Figure 1:**
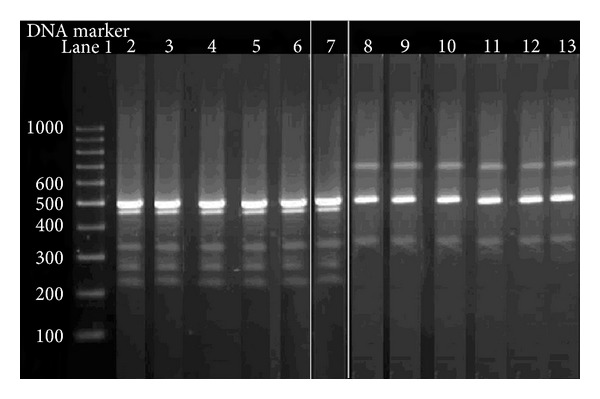
Random-amplified PCR of genomic DNA of two different snail strains (resistant and susceptible) of *Biomphalaria alexandrina *with arbitrary primer OPB-18 (5′-CCACAGCAGT-3′). Lane 1: 100 bp DNA ladder and lanes 2–7: individual of resistant snails and lanes 8–13: individual of susceptible *B. alexandrina*. The lanes from 1 to 6 and from 8 to 13 were on the same gel run, but lane 7 was on a separate one.

**Figure 2:**
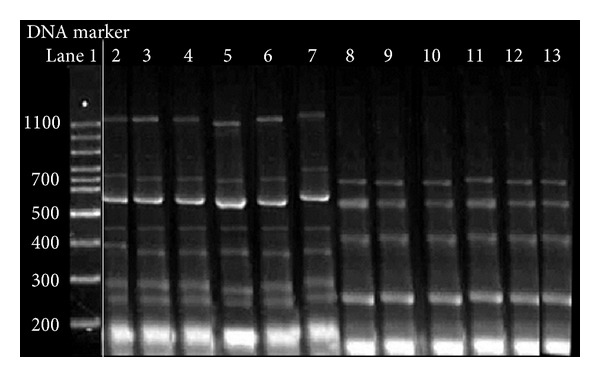
Random-amplified PCR of genomic DNA of two different snail strains (resistant and susceptible) of *Biomphalaria alexandrina *with arbitrary primer OPC-11 (5′-AAAGCTGCGG-3′). Lane 1: 100 bp DNA ladder, lanes 2–7; individuals of resistant snails, and lanes 8–13: individuals of susceptible *B. alexandrina*. The lanes from 1 to 13 were on the same gel run, and the space between the marker and lane 2 was wide, so it was cut and the lanes were spliced.

The genomic DNA amplified by using the primer OPA-02 (5′-TGCCGAGCTG-3′) gave one polymorphic band of approximately 730 bp in the susceptible strains only and three differentiating bands of nearly 330, 430, and 670 bp in resistant strains. As shown in Figure 1, the genomic DNA amplified by using the primer OPB-18 (5′-CCACAGCAGT-3′) presented a polymorphic band being approximately 700 bp in the susceptible strains only. In resistant strains, the primer yielded three differentiating bands of nearly 230, 260, and 470 bp. The results obtained from the electrophoresis on agarose gel of the amplified products of genomic DNA with the primer OPC-11 (5′-AAAGCTGCGG-3′) showed a number of differentiating bands in both susceptible and resistant strains. The genomic DNA, which was amplified with this primer, presented with a specific band of 400 bp in susceptible lineages and four bands of approximately 290, 380, 540, and 1010 bp in the resistant lineages (Figure 2). One band only of 400 bp in the susceptible linage and another one of 260 bp in the resistant linage were obtained using the OPD-10 (5′-GGTCTACACC-3′) primer. The amplified genomic DNA with the primer OPD-18 (5′-GAGAGCCAAC-3′) presented with two differentiating bands of nearly 230 and 1000 bp in the susceptible lineages and three specific bands of 150, 300, and 500 bp in resistant lineages.

## 4. Discussion

The infection rate obtained in the present study demonstrates that *B. alexandrina* is an efficient intermediate host for *S. mansoni* and consequently plays an important role in schistosomiasis transmission in Egypt.

The RAPD-PCR technique was used in the current work to determine the susceptibility/resistant genetic variability of *B. alexandrina* snails. Eight ten base pair (bp) oligonucleotide primers were each added to an individual sample of DNA, which was then subjected to PCR. The resulting amplified DNA bands were polymorphic segments with different band sizes depending upon the genomic DNA and the primer. The RAPD-PCR products were analyzed using agarose gel, which represents an effective and cheap method [33, 35]. However, other authors have used the alternative polyacrylamide gel electrophoresis method [21, 33, 36].

The results of the current work reveal the low coefficient of similarity between the susceptible and resistant snails studied (Table 1). This may be due to the short period (one year) allotted for snail rearing in the laboratory. In a study by Spada et al. [26] in which snails were reared for 20 years in the laboratory, the authors yielded a high coefficient of similarity. These authors expected low genetic variability of their strains and concluded that laboratory strains undergo much less intense selective pressures than the field isolates have.

Of the eight primers used in the present study, three primers, namely, OPA-01, OPA-06, and OPM-04, whose sequence of oligonucleotides are (5′-CAGGCCCTTC-3′), (5′-GGTCCCTGAC-3′) and (5′-GGCGGTTGTC-3′), respectively, were unable to detect polymorphic markers between the resistant and susceptible lines of *B. alexandrina *snails. However, these primers were efficient in distinguishing genetic variability of the resistant and susceptible lines of *B. glabrata* and *B. tenagophila* [24, 26, 32, 33]. These primers might amplify very restricted areas of the genomes of *B. glabrata* and *B. tenagophila* specific for resistance or susceptibility, which are not present in* B. alexandrina *snails. Interestingly, one of these primers, the OPA-01 primer, resulted in high genetic variability between *B. alexandrina *strains from different provinces in Egypt using the RAPD-PCR technique [37]. The present study showed that with five out of the eight utilized primers, specific bands of different lengths were amplified in the resistant and susceptible phenotype snails. To our knowledge, four of the five utilized primers (OPB-18, OPC-11, OPD-10, and OPD-18) had not yet been studied with *Biomphalaria spp*. The fifth primer OPA-02 (5′-TGCCGAGCTG-3′) had been tried previously with different *Biomphalaria* species [21, 25, 26]. In the current study, this primer resulted in three polymorphic bands with nearly 330, 430, and 680 bp in the resistant strains and one band with 730 bp in the susceptible strain. Abdel-Hamid et al. [21] used the same primer on* B. alexandrina *originating from the Delta region of Egypt to determine markers of resistance. They obtained only one band with 430 bp in the resistant strains, which we also obtained in the current study. The OPA-02 primer was also used by Abdel-Hamid et al. [25] and Spada et al. [26] for the analysis of the genetic variability between resistant and susceptible lineages of *B. tenagophila* and *B. glabrata*. Using this primer, the authors were unable to detect any polymorphic markers between resistant and susceptible lines. This marker may be a specific marker distinguishing the resistant and susceptible *B. alexandrina* from other species.

In the current work, we searched for primers capable of differentiating susceptible and resistant strains. As mentioned above, five of the eight utilized primers were able to amplify specific bands of different lengths between the resistant and susceptible phenotype snails. In addition to the OPA-02 primer, which was used in previous studies, we tried four random primers that, to our knowledge, have not been used before with *Biomphalaria* species. These primers are OPB-18 (5′-CCACAGCAGT-3′), OPC-11 (5′-AAAGCTGCGG-3′), OPD-10 (5′-GGTCTACACC-3′), and OPD-18 (5′-GAGAGCCAAC-3′). Each of these primers was able to demonstrate one or more differentiating polymorphic bands in the resistant and susceptible strains in *B. alexandrina*. 

We found that the detection of susceptible and resistant bands using the RAPD-PCR technique is a suitable and efficient methodological approach for the analysis of genetic variability among schistosomiasis intermediate hosts. However, future studies should sequence the PCR products to predict the function of the identified DNA sequences and to assist in explaining the levels of polymorphism reported between the susceptible and resistant snails.

As has been concluded by other researchers, the RAPD-PCR technique was found to be sensitive, fast, and easy to perform [32, 38]. Using the RAPD technique with the abovementioned primers can identify genomic markers that are specifically related to the *B. alexandrina*/*S. mansoni* relationship in the absence of specific nucleotide sequence information. Consequently, this approach can be used in epidemiologic surveys investigating the genetic diversity of *B. alexandrina* snails, as it is a time-saving technique when compared to the conventional parasitological methods. The ability to determine resistant markers in *B. alexandrina *snails could pave the way for further studies based on the use of refractory snails as a means by which to biologically control the transmission of disease, although this issue remains a matter of discussion. However, the use of this approach in concert with other measures could create new strategies for the control of schistosomiasis transmission.

## Figures and Tables

**Table 1:** Dice's similarity coefficient (*S*) between susceptible and resistant strains of *Biomphalaria alexandrina *snails.

	OPA-2	OPB-18	OPC-11	OPD-10	OPD-18
Number of shared bands (*a*)	2	2	3	2	1
Number of bands in susceptible not in resistant strain (*b*)	1	1	1	1	2
Number of bands in resistant not in susceptible strain (*c*)	3	3	4	1	3
Similarity coefficient (*S*)	50%	50%	55%	66%	29%

*S* = 2*a*/2*a* + *b* + *c*.
